# Renalase attenuates hypertension, renal injury and cardiac remodelling in rats with subtotal nephrectomy

**DOI:** 10.1111/jcmm.12813

**Published:** 2016-02-29

**Authors:** Jianyong Yin, Zeyuan Lu, Feng Wang, Zhenzhen Jiang, Limin Lu, Naijun Miao, Niansong Wang

**Affiliations:** ^1^ Department of Nephrology and RheumatologyShanghai Jiao Tong University Affiliated Sixth People's HospitalShanghaiChina; ^2^ Department of Physiology and PathophysiologyShanghai Medical CollegeFudan UniversityShanghaiChina

**Keywords:** renalase, cardiorenal syndromes, subtotal nephrectomy, renal dysfunction, cardiac remodelling

## Abstract

Chronic kidney disease is associated with higher risk of cardiovascular complication and this interaction can lead to accelerated dysfunction in both organs. Renalase, a kidney‐derived cytokine, not only protects against various renal diseases but also exerts cardio‐protective effects. Here, we investigated the role of renalase in the progression of cardiorenal syndrome (CRS) after subtotal nephrectomy. Sprague–Dawley rats were randomly subjected to sham operation or subtotal (5/6) nephrectomy (STNx). Two weeks after surgery, sham rats were intravenously injected with Hanks' balanced salt solution (sham), and STNx rats were randomly intravenously injected with adenovirus‐β‐gal (STNx+Ad‐β‐gal) or adenovirus‐renalase (STNx+Ad‐renalase) respectively. After 4 weeks of therapy, Ad‐renalase administration significantly restored plasma, kidney and heart renalase expression levels in STNx rats. We noticed that STNx rats receiving Ad‐renalase exhibited reduced proteinuria, glomerular hypertrophy and interstitial fibrosis after renal ablation compared with STNx rats receiving Ad‐β‐gal; these changes were associated with significant decreased expression of genes for fibrosis markers, proinflammatory cytokines and nicotinamide adenine dinucleotide phosphate (NADPH) oxidase components. At the same time, systemic delivery of renalase attenuated hypertension, cardiomyocytes hypertrophy and cardiac interstitial fibrosis; prevented cardiac remodelling through inhibition of pro‐fibrotic genes expression and phosphorylation of extracellular signal‐regulated kinase (ERK)‐1/2. In summary, these results indicate that renalase protects against renal injury and cardiac remodelling after subtotal nephrectomy *via* inhibiting inflammation, oxidative stress and phosphorylation of ERK‐1/2. Renalase shows potential as a therapeutic target for the prevention and treatment of CRS in patients with chronic kidney disease.

## Introduction

Recently, it has been increasingly recognized that primary disorder of kidney will induce or worsen pathological injuries in heart, which in turn can lead to accelerated organ dysfunction in both systems. This phenomenon has been defined as chronic renocardiac syndrome or type 4 cardiorenal syndrome (CRS) 1. Clinical studies demonstrated that patients with primary chronic kidney disease (CKD) show higher incidence of cardiovascular diseases (CVD) and a 10‐ to 20‐fold increased risk of cardiac death compared with that of the general population 2, 3. However, there are still no effective strategies for treatment of CRS for now. Thus, it is urgent to identify underlying mechanisms of CRS progression for developing novel therapy.

Although the exact pathophysiology mechanisms underlying this bidirectional cardiorenal crosstalk are not fully elucidated yet, traditional risks in CKD such as humoural and haemodynamic disturbance and activation of renin–angiotensin–aldosterone system and sympathetic nerve system (SNS) may contribute to the pathogenesis of CVD 4, 5. Recently, it has been suggested that abnormalities in bone‐mineral axis and deficiency of erythropoietin (EPO) or klotho may directly aggravate cardiac hypertrophy and participate in the pathogenesis of worsening of renal function and cardiovascular complication 6, 7. Therefore, special emphasis should be placed on the endocrine function of kidney 8. The reduction or loss of EPO, calcitriol or klotho and yet‐to‐be known kidney‐derived hormones or cytokines as a result of renal dysfunction may be potential mechanisms of CRS 9.

Renalase, a recently discovered flavoprotein from kidney, can regulate blood pressure by degrading circulatory catecholamines 10. Our previous reports demonstrated that renalase is up‐regulated during various stresses including hypoxia, ischaemia/reperfusion and oxidative stress 11, 12, 13. Administration of recombinant renalase protects against AKI, contrast‐induced nephropathy and cardiac ischaemia/reperfusion injury 12, 13, 14, 15. Clinical studies showed that renalase deficiency or single‐nucleotide polymorphisms are associated with essential hypertension, cardiac hypertrophy, stroke and diabetes 16, 17, 18, 19, 20. Recently, mounting evidence suggested that renalase exerts its cytoprotective effects by interacting with its plasma membrane receptor other than metabolizing catecholamines 21, 22, 23. Thus, renalase may serve as a cytokine that regulates kidney function in autocrine or paracrine manner 24. Nevertheless, whether renalase supplementation could prevent the progression of renal injury and occurrence of remote organ injury in CKD and its mechanisms still remain unknown.

Western blot analyses demonstrated that plasma renalase concentration in CKD patients is substantially decreased in comparison to healthy population 10. However, several clinical studies using ELISA reported opposite results 25, 26. These opposite results may be inaccurate because of a not validated antibody used in the commercially kit which would non‐specifically recognize renalase breakdown products or unrelated epitopes 27. Consistently, renalase deficiency is also present in rats subjected to subtotal nephrectomy 28. Thus, it is reasonable to speculate that renalase may be also a crucial kidney‐derived modulator for CRS progression. In present study, we examined the effects of renalase supplementation by an adenovirus delivery on the progression of type 4 CRS in a rat subtotal nephrectomy model. Furthermore, the potential mechanisms mediating renalase's protective effects were also investigated.

## Material and methods

### Construction of adenovirus vectors

The replication‐deficient recombinant adenovirus vectors expressing the rat renalase mRNA sequence or β‐galactosidase (β‐gal) were generated respectively, under the control of the cytomegalovirus enhancer/promoter. These adenoviruses were amplified in HEK‐293A cells and purified by CsCl ultracentrifugation and store at −80°C in Hanks' balanced salt solution (HBSS) with a concentration of 1.0 × 10^10^ plaque formation unit (PFU)/ml.

### Animal

All the animal experiments were approved by the Animal Care and Ethics Committee of Shanghai Jiao Tong University Affiliated Sixth People's Hospital. Male Sprague–Dawley rats were provided by Shanghai Science Academy animal center. All the rats were housed in a 12/12 hrs light/dark cycle with free access to water and fed with standard rat chow.

### Rat model of CKD and experimental protocols

Male Sprague–Dawley rats weighing 200–250 g, after 7‐day adaption period, were randomly allocated to either sham‐operated group or 5/6 subtotal nephrectomy group. The animals were anaesthetized by intraperitoneally injection of sodium pentobarbital (50 mg/kg) and two‐stage subtotal nephrectomy was performed as previously described 29. Briefly, the upper and lower poles of the left kidney were resected and bleeding was controlled. One week later, the right kidney was removed after ligation of the renal blood vessels and the ureter. The sham‐operated rats underwent the same procedures, but only the envelope capsule was removed.

Two weeks after surgery, the rats were further randomly divided into following groups: (*i*) sham‐operated treated with tail vein injection of 1.2 ml HBSS (sham, *n* = 10); (*ii*) subtotal nephrectomy treated with tail vein injection of 1.2 × 10^10^ PFU control adenovirus (STNx+Ad‐β‐gal, *n* = 10); (*iii*) subtotal nephrectomy treated with tail vein injection of 1.2 × 10^10^ PFU adenovirus‐renalase (STNx+Ad‐renalase, *n* = 10). The effect of renalase gene delivery on CRS was assessed 4 weeks after adenovirus injection (corresponding to Week 6 of CKD). At the end of the experiment, all the rats were kept 24 hrs in metabolic cages for urine collection and echocardiography was also examined during the last week. Then all the animals were killed and blood as well as kidney and heart tissues were harvested for analyses.

### Renal function assessment

Blood samples were collected from abdominal aorta and centrifuged to obtain plasma and then separated into aliquots and stored at −20°C. Blood urea nitrogen (BUN) and urinary creatinine and proteinuria were measured by commercial kit (Nanjingjiancheng, Nanjing, Jiangsu, China). The ratio of left kidney weight to bodyweight (bw) was also calculated to evaluate renal dysfunction. Plasma norepinephrine concentration was evaluated by ELISA kit (LDN, Nordhorn, Germany).

### Systolic blood pressure measurement

Systolic blood pressure (SBP) was measured prior to adenovirus injection and once a week after gene delivery by a tail‐cuff method, using CODA blood pressure systems (Kent Scientific, Torrington, CT, USA). Prior to the actual experiments, the conscious animals were training for three consecutive days to become accustomed to the procedure. For each animal, at least 15 measurements were recorded to calculate a mean blood pressure and heart rate.

### Cardiac functional assessment

Transthoracic echocardiography was performed at the end‐point using a VeVo770 High Resolution Imaging System (Visual Sonics Inc., Toronto, ON, Canada) with 17.5 MHz transducer as previously described 30. M‐mode and 2D parasternal short‐axis views were analysed in the anaesthetized rats by trained echocardiographers who were blinded to the treatments.

Before being killed, all the rats underwent LV (left ventricular) catheterization to assess haemodynamics changes as previously described 31. The transducer was attached to RM6240BD multichannel biotic signal collection and analysis instrument (Taimeng, Chengdu, China) to determine cardiac function. LV systolic pressure, LV end‐diastolic pressure (LVEDP) and the maximal rate of pressure rise (dP/dt_max_) and fall (dP/dt_min_) were recorded respectively.

Heart weight (HW) index were analysed among the three groups. The hearts were rapidly excised, cleaned and weighed. The left ventricles were separated and weighed. The ratios of the whole HW to bw, the ratios of the LV weight (LW) to bw were calculate to evaluate cardiac hypertrophy.

### Histology analysis

All the tissues were fixed in 4% paraformaldehyde and embedded in paraffin. Tissue sections (4–6 μm) were stained with haematoxylin and eosin, Masson trichrome solution or Sirus Red to assess histological injury and fibrosis.

A semi‐quantitative score was used to determine the severity of glomerulosclerosis and tubulointerstitial injury as described previously 32, 33. Glomerular area was measured as described previously 34. All evaluation was performed by an observer who was blind to the experimental protocol. To evaluate the degree of fibrosis, 10 non‐overlapping fields of each section were randomly chosen. Cross‐section area of cardiomyocytes was calculated from the average of 50 cardiomyocytes per section as described previously 35.

For immunohistochemistry, kidney sections were stained with antirat monoclonal antibodies against CD68 (Sigma‐Aldrich, St. Louis, MO, USA), CD86, CD163 and renalase (both from Abcam, Cambridge, MA, USA) as previously described 36. All the measurements were calculated by ImagePro Plus Systems.

### Quantitative real‐time PCR

Total RNA were extracted from the renal cortex or LV tissue using Trizol (Invitrogen, Carlsbad, CA, USA) and was reverse transcribed into cDNA with M‐MLV Reverse Transcriptase (Promega, Madison, WI, USA). Real‐time PCR was performed with SYBE Green PCR master Mix (Tarkara, Dalian, China) using StepOnePlus PCR Systems (Applied Biosystems, Foster City, CA, USA) according to the manufacturer's protocol. Quantitation was normalized to internal control GAPDH and 2^−ΔΔCT^ method was used to determine relative gene expression levels. All the primer pairs are seen in Table S1.

### Western blot analysis

Total protein was prepared from frozen tissues by homogenization. Protein concentrations were determined by BCA assay (Beyotime, Suzhou, Jiangsu, China) and protein samples were separated by 10–12% SDS‐PAGE and then transferred to polyvinylidene difluoride membrane. The membranes were blocked with 5% non‐fat dried milk and then incubated with primary antibodies against matrix metalloproteinase 1 (MMP‐1), tissue inhibitor of metalloproteinase‐1 (TIMP‐1), renalase (Abcam), phospho‐ERK‐1/2, total ERK‐1/2, phospho‐p38, total p38 (Cell Signaling Technology, Danvers, MA, USA). Horseradish peroxidase‐conjugated secondary antibodies (Beyotime) were used and visualized by Image Quant LAS 4000 Mini System (GE Healthcare, Pittsburgh, PA, USA). The bands were analysed using Image J software and GAPDH or tubulin (Proteintech, Chicago, IL, USA) was used as internal control.

### Statistical analyses

SPSS software 19.0 (IBM, Armonk, NY, USA) was used for all the statistical analyses. All the values are expressed as mean ± S.E.M. One‐way anova followed by a with Tukey's Multiple Comparison Test was used to compare parametric data while Kruskal–Wallis test followed by the Mann–Whitney *U*‐test was used for non‐parametric data comparison. A value of *P* < 0.05 were considered statistically significant.

## Results

### Adenovirus‐mediated gene delivery efficacy

As shown in Figure S1, subtotal nephrectomy resulted in dramatic decrease in renalase expression both in the plasma, remnant kidney and heart compared with sham group. Renalase gene delivery restored renalase expression nearly to normal levels in circulation, kidney and heart according to Western blot and PCR results in STNx rats (Fig. S1). Consistently, immunohistochemistry analysis also showed adenovirus‐renalase effectively increase the percentage of renalase‐positive cells in kidney compared with Ad‐β‐gal‐treated STNx rats (Fig. S2).

### Renalase gene delivery ameliorated renal dysfunction in STNx rats

Compared to sham group, STNx rats exhibited significant renal dysfunction characterized by increased plasma BUN, urine 24 hrs total protein levels, and decreased creatinine clearance as shown in Table 1. After 4 weeks of treatment, systemic delivery of renalase significantly decreased plasma BUN and proteinuria in STNx rats. Kidney weight/bw was also lower in Ad‐renalase‐treated group than Ad‐β‐gal‐treated group (Table 1). In addition, Ad‐renalase delivery attenuated creatinine clearance decline in STNx rats. Furthermore, plasma norepinephrine level in Ad‐renalase‐treated STNx rats was also significantly lower than Ad‐β‐gal‐treated STNx rats (Table 1); suggesting renalase may participate in the metabolism of catecholamines.

**Table 1:** General characteristics of rats and renal function assessments 6 weeks after surgery

	Sham	STNx+Ad‐β‐gal	STNx+Ad‐renalase
Bodyweight (g)	485 ± 17	410 ± 19*	425 ± 15
Left kidney weight/bw (mg/g)	5.31 ± 0.17	4.5 ± 0.48**	4.1 ± 0.39** ^,^ #
Serum urea nitrogen	5.62 ± 0.86	17.23 ± 1.21**	13.59 ± 0.99* ^,^ #
Proteinuria (mg/24 hrs)	12.08 ± 1.29	134 ± 9.76***	59.90 ± 5.79*** ^,^ ##, #
Creatinine clearance (ml/min.)	1.56 ± 0.18	0.87 ± 0.20**	1.13 ± 0.2* ^,^ #
Norepinephrine (pg/ml)	438 ± 49	1280 ± 81*	667 ± 78#

*P < 0.05 *versus* sham; ^#^ *P* < 0.05 *versus* STNx+Ad‐β‐gal; **P < 0.01 *versus* sham; ^##^ *P* < 0.01 *versus* STNx+Ad‐β‐gal; ***P < 0.001 *versus* sham; ^###^ *P* < 0.001 *versu*s STNx+Ad‐β‐gal.

All values are presented as means ± S.E.M. (*n* = 10).

STNx: subtotal nephrectomy; bw: bodyweight.

### Renalase supplementation attenuated renal pathological injury

**Figure 1:**
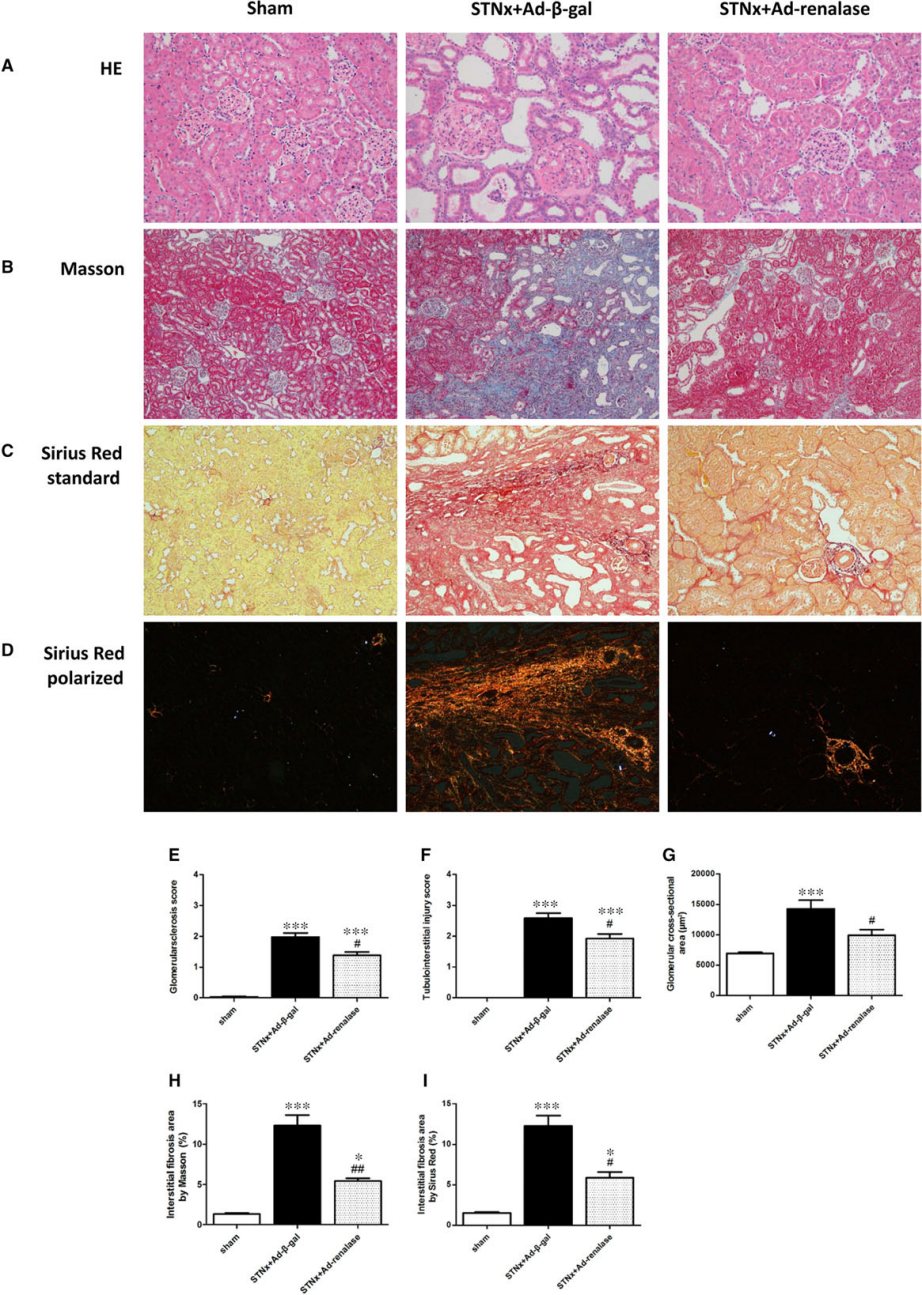
Renalase gene delivery attenuated renal injury 6 weeks after subtotal nephrectomy. (**A**) Histologic examination of renal injury by HE staining for sham‐operated, Ad‐β‐gal ‐treated STNx rats and Ad‐renalase‐treated STNx rats, magnification, ×200. (**B**) Assessment of interstitial fibrosis by Masson staining, magnification, ×100. (**C**) Representative picture of Sirus Red staining in standard light, magnification, ×100. (**D**) Representative picture of Sirus Red staining in polarized light, magnification, ×100. (**E**) Semi‐quantitative analysis of glomerular injury. (**F**) Semi‐quantitative analysis of tubulointerstitial injury. (**G**) Quantification of glomerular cross‐section area. (**H**) Quantitative analysis of interstitial fibrosis by Masson. (**I**) Quantitative analysis of interstitial fibrosis by Sirus Red. All values are presented as means ± S.E.M. (*n* = 10). *P < 0.05 versus sham; ***P < 0.001 *versus* sham; #*P* < 0.05 *versus* STNx+Ad‐β‐gal; ##*P* < 0.01 *versus* STNx+Ad+β‐gal.

Kidney sections from STNx rats revealed a remarkable glomerular hypertrophy and tubular enlargement (Fig. 1A). Ad‐renalase treatment significantly decreased glomerular area (Fig. 1G) and ameliorated glomerularsclerosis and tubulointerstitial injury after subtotal nephrectomy as assessed by pathological score (Fig. 1E and F). To evaluate the extent of interstitial fibrosis, kidney tissues were stained with Masson and Sirus Red. Results showed that renal interstitial fibrosis in Ad‐renalase‐treated rats was less severe than that in Ad‐β‐gal‐treated STNx rats as shown in Figure 1B–D. Consistent with the histological data, mRNA levels of fibrosis markers, including collagen I, collagen III and transforming growth factor (TGF)‐β1 were significantly higher in the remnant kidneys of Ad‐β‐gal‐treated STNx rats than in those of sham rats; and Ad‐renalase treatment significantly decreased mRNA expression of these fibrosis markers (Fig. 3A).

### Renalase inhibited inflammation and oxidative stress in the remnant kidney

**Figure 2:**
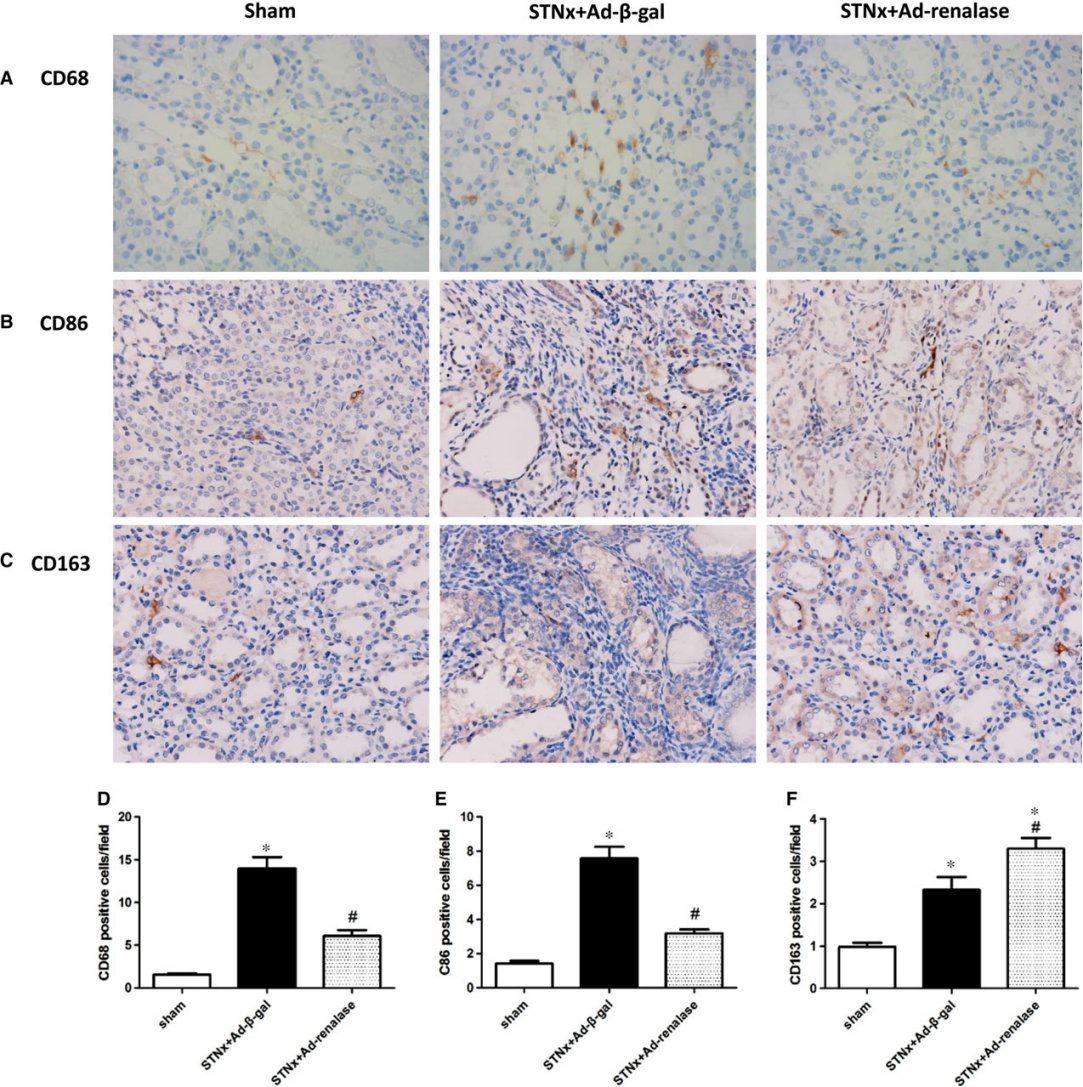
Renal macrophage M1/M2 phenotype was altered by renalase supplementation. (**A**) Representative photographs of immune staining for CD68, magnification, ×400. (**B**) Representative photographs of CD86 immunostaining, magnification, ×400. (**C**) Representative photographs of CD163 immunostaining, magnification, ×400. Quantitative analysis of CD68 (**D**), CD86 (**E**) and CD163 (**F**) positive macrophage numbers per field. All values are presented as means ± S.E.M. (*n* = 10). *P < 0.05 *versus* sham; ^#^ *P* < 0.05 *versus* STNx+Ad‐β‐gal.

To explore whether renalase was involved in macrophage infiltration, activation and polarization, we examined renal infiltration of total macrophage (CD68), M1‐like (CD86) and M2‐like macrophage (CD163). Immunohistochemistry analysis showed that CKD rats displayed increased all subtypes of macrophage infiltration and enhanced M1 polarization. Ad‐renalase administration significantly diminished total macrophage infiltration numbers especially M1 numbers than Ad‐β‐gal‐treated rats after subtotal nephrectomy (Fig. 2). In addition, Ad‐renalase‐treated rats exhibited slightly more M2 infiltration than Ad‐β‐gal‐treated rats.

**Figure 3:**
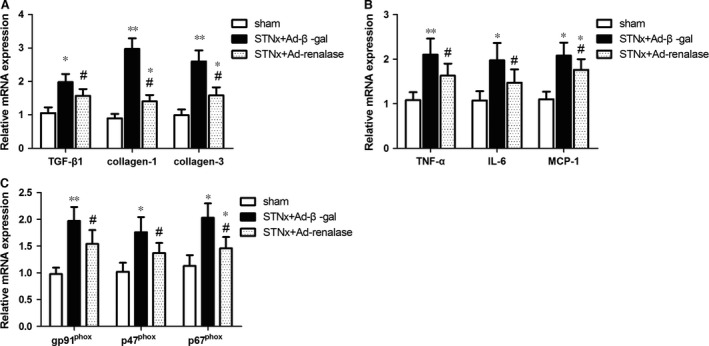
Ad‐renalase‐treated rats exhibited decreased mRNA expression of fibrosis markers, pro‐inflammatory cytokines and NADPH oxidative stress components in the kidney. (**A**) Relative mRNA expression collagen I, collagen III and TGF‐β1. (**B**) Relative mRNA expression of TNF‐α, IL‐6 and MCP‐1. (**C**) Relative mRNA expression of gp91^phox^, P47^phox^ and P67^phox^. All values are expressed as mean ± S.E.M. (*n* = 10). *P < 0.05 *versus* sham; ^#^ *P* < 0.05 *versus* STNx+Ad‐β‐gal; **P < 0.01 *versus* sham.

To determine the effects of renalase supplementation on inflammation and oxidative stress, mRNA levels of pro‐inflammatory cytokines and NADPH oxidase components were also measured by real‐time PCR. Nephrectomized rats showed higher expression levels of pro‐inflammatory cytokines, including tumour necrosis factor (TNF)‐α, interleukin (IL)‐6, monocyte chemotactic protein (MCP)‐1 (Fig. 3B) and NADPH oxidase components, including gp91^phox^, p47^phox^ and p67^phox^ compared with sham (Fig. 3C); and Ad‐renalase treatment decreased the expression of pro‐inflammatory cytokines and NADPH oxidase components. Taking together, these findings indicated renalase supplementation might protect renal injury after renal ablation *via* inhibiting inflammation activation and oxidative stress in remnant kidney.

### Effects of renalase on systolic blood pressure

**Figure 4:**
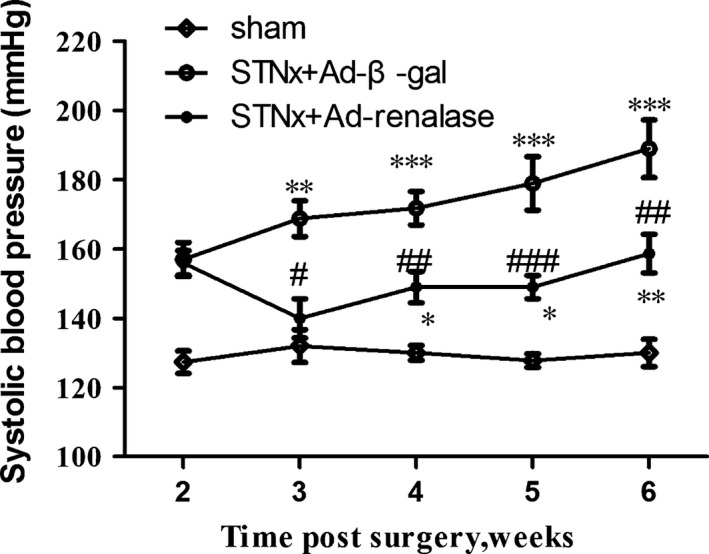
Systolic blood pressure of sham‐operated, Ad‐β‐gal‐treated STNx rats and Ad‐renalase‐treated STNx rats. Systolic blood pressure values are expressed as mean ± S.E.M. (*n* = 10). *P < 0.05 *versus* sham; ^#^ *P* < 0.05 *versus* STNx+Ad‐β‐gal; **P < 0.01 *versus* sham; ^##^ *P* < 0.01 *versus* STNx+Ad‐β‐gal; ***P < 0.001 *versus* sham; ^###^ *P* < 0.001 *versus* STNx+Ad‐β‐gal.

As shown in Figure 4, SBP in STNx rats gradually increased during the observation compared with the sham group and Ad‐renalase delivery significantly decreased SBP in STNx rats compared with Ad‐β‐gal‐treated rats.

### Renalase supplementation prevented cardiac remodelling in STNx rats

The HW/bw and LV weight/bw were significantly higher in STNx rats than the sham and significantly lower in Ad‐renalase‐treated rats than the Ad‐β‐gal‐treated rats (Table 2). Concomitant alterations were found in echocardiography (Table 2 and Fig. S3), 4 weeks of treatment with Ad‐renalase significantly decreased LVPWd, LVAWd and increased LVIDs and LVIDs (Table 2). However, there was no difference in FS and EF among the three groups.

**Table 2:** Cardiac function assessment 6 weeks after surgery

	Sham	STNx+Ad‐β‐gal	STNx+Ad‐renalase
HR	371 ± 31	410 + 13	402 ± 19
HW/bw (mg/g)	2.35 ± 0.17	3.9 ± 0.25***	3.68 ± 0.21** ^,^ #
LW/HW (mg/g)	4.1 ± 0.36	6.1 ± 0.53***	5.6 ± 0.49** ^,^ #
Echocardiography			
LVPWd (mm)	2.01 ± 0.08	2.88 ± 0.16***	1.99 ± 0.13###
LVPWs (mm)	3.22 ± 0.16	4.24 ± 0.08***	3.60 ± 0.15##
LVAWd (mm)	1.81 ± 0.07	2.43 ± 0.01***	1.91 ± 0.08##
LVAWs (mm)	3.10 ± 0.15	4.22 ± 0.13***	3.64 ± 0.13* ^,^ #
LVIDd (mm)	7.05 ± 0.27	5.57 ± 0.20*	7.05 ± 0.31##
LVIDs (mm)	3.32 ± 0.40	1.58 ± 0.19**	2.66 ± 0.29##
Fractional shortening (%)	52.02 ± 3.57	71.34 ± 3.26	60.59 ± 3.64
EF (%)	94.35 ± 1.6	80.81 ± 3.39	87.72 ± 2.7
Cardiac catheterization			
LVEDP (mmHg)	2.3 ± 0.49	6.5 ± 0.56**	4.5 ± 0.40* ^,^ #
LVESP (mmHg)	70.45 ± 5.98	69.43 ± 7.89	80.32 ± 5.76
+dP/dt_max_ (mmHg/sec.)	4954 ± 389	5130 ± 447	4986 ± 450
−dP/dt_min_ (mmHg/sec.)	−4823 ± 430	−3960 ± 536	−4423 ± 378

*P < 0.05 *versus* sham; ^#^ *P* < 0.05 *versus* STNx+Ad‐β‐gal; **P < 0.01 *versus* sham; ^##^ *P* < 0.01 *versus* STNx+Ad‐β‐gal; ***P < 0.001 *versus* sham; ^###^ *P* < 0.001 *versus* STNx+Ad‐β‐gal.

All values are presented as means ± S.E.M. (*n* = 8).

LVPWd: LV end‐diastolic posterior wall thickness; LVPWs: LV end‐systole posterior wall thickness; LVAWd: LV end‐diastolic anterior wall thickness; LVAWs: LV end‐systole anterior wall thickness; LVIDd: LV internal diastolic diameter; LVIDs: LV internal systolic diameter; dP/dtmax: rate of LV pressure rise; −dP/t_min_: rate of LV pressure fall; LVEDP: LV end‐diastolic pressure; LVESP: LV end‐systolic pressure.

**Figure 5:**
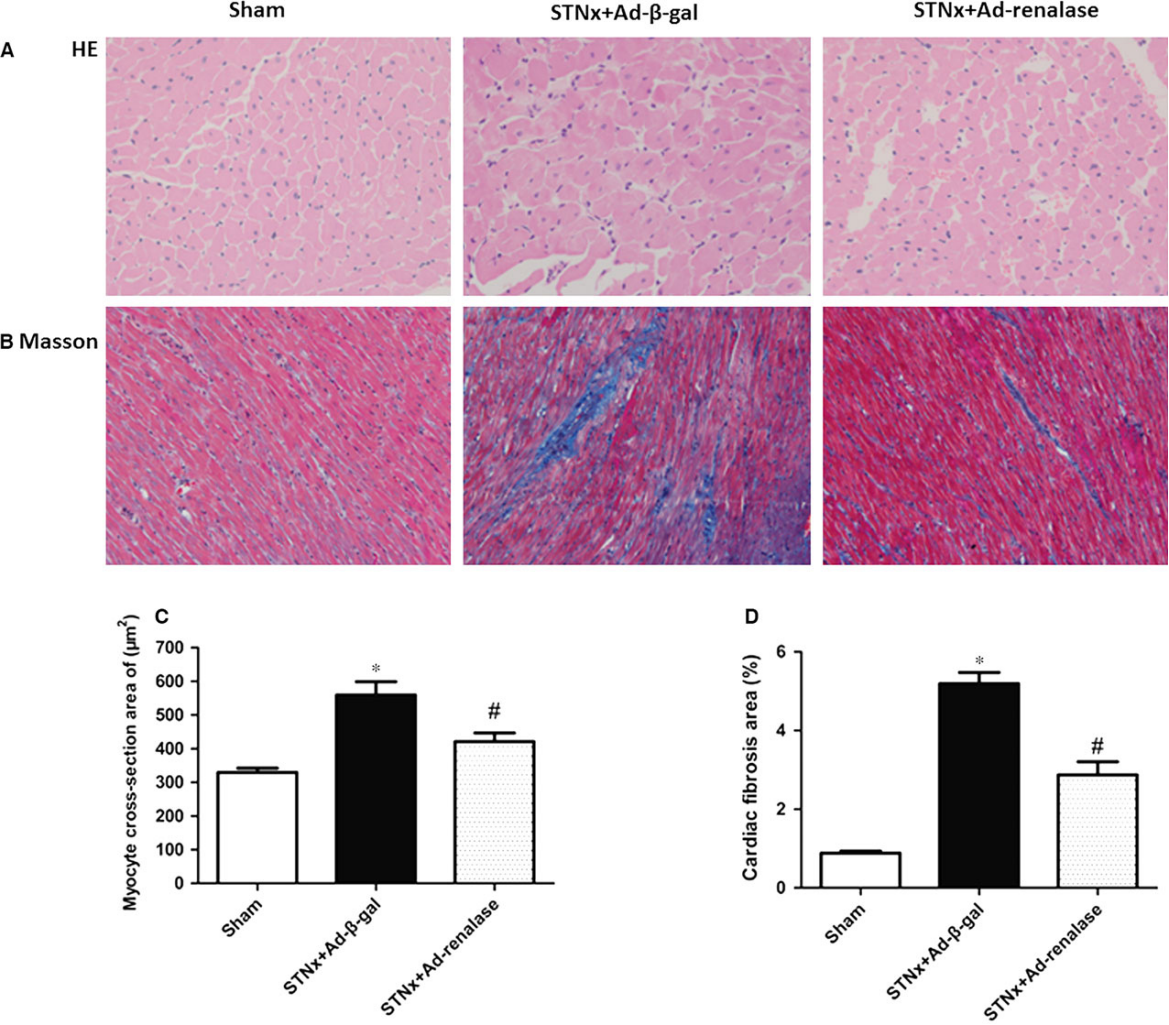
Systemic delivery of renalase ameliorated cardiomyocytes hypertrophy and cardiac fibrosis. (**A**) Representative photographs of HE of the heart sections from three groups at week 6, magnification, ×400. (**B**) Representative photographs of Masson staining for cardiac fibrosis, magnification, ×200. (**C**) Quantitative analysis of cross‐section area of cardiomyocytes. (**D**) Quantitative evaluation of cardiac fibrosis. All values are presented as means ± S.E.M. (*n* = 10). *P < 0.05 *versus* sham; ^#^ *P* < 0.05 *versus* STNx+Ad‐β‐gal.

**Figure 6:**
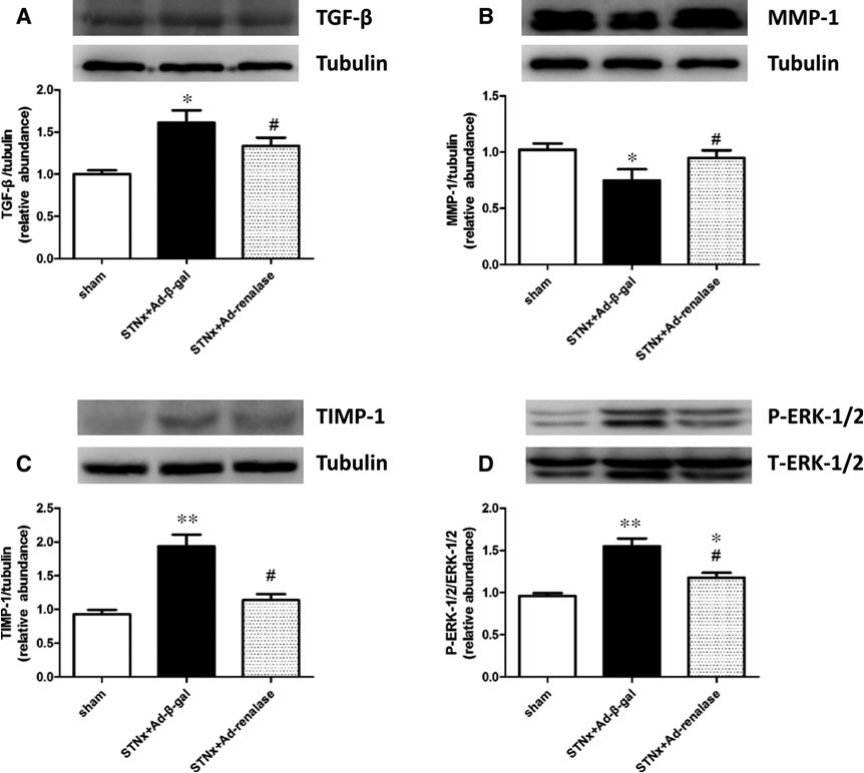
Ad‐renalase treatment normalized cardiac expression of pro‐fibrotic markers and phosphorylated ERK‐1/2 in CKD rats. Representative Western blot and quantification of TGF‐β (**A**), MMP‐1 (**B**), TIMP‐1 (**C**) and phosphorylated ERK‐1/2 (**D**) expression in left ventricle from three groups at week 6. All values are presented as means ± S.E.M. (*n* = 10). *P < 0.05 *versus* sham; **P < 0.01 *versus* sham; ^#^ *P* < 0.05 *versus* STNx+Ad‐β‐gal.

Histological sections of hearts demonstrated that subtotal nephrectomy led to elevated cross‐section area of cardiomyocytes and Ad‐renalase delivery ameliorated cardiac cardiomyocytes hypertrophy in STNx rats (Fig. 5A and C). Cardiac fibrosis assessment, determined by Masson staining, showed that STNx rats had more matrix deposition than sham; and lesser cardiac interstitial fibrosis was observed in the Ad‐renalase‐treated rats compared with Ad‐β‐gal‐treated STNx rats (Fig. 5B and D). In addition, Western blot showed that expression of MMP‐1 was significantly lower in Ad‐β‐gal‐treated rats than sham and significantly higher in Ad‐renalase‐treated rats than Ad‐β‐gal‐treated rats. Meanwhile, the expression of TIMP‐1 and TGF‐β was significantly higher in Ad‐β‐gal‐treated rats than sham and significantly lower in Ad‐renalase‐treated rats compared with Ad‐β‐gal‐treated rats (Fig. 6A–C).

### Deactivation of mitogen‐activated protein kinases after treatment with Ad‐renalase

**Figure 7:**
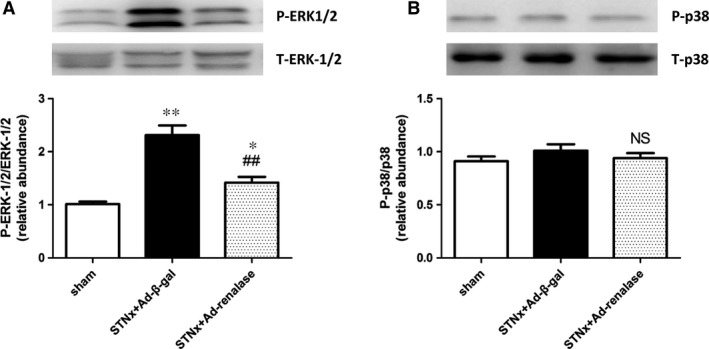
Ad‐renalase treatment suppressed ERK‐1/2 phosphorylation in the remnant kidney. Representative Western blots of phosphorylated ERK‐1/2 (**A**) and phosphorylated p38 (**B**) in kidney. Values are presented as means ± S.E.M. (*n* = 10). *P < 0.05 *versus* sham; **P < 0.01 *versus* sham; ^##^ *P* < 0.01 *versus* STNx+Ad‐β‐gal; NS, no statistically significant.

The phosphorylated of ERK‐1/2 (p‐ERK‐1/2) in heart (Fig. 6D) and kidney (Fig. 7A) was both significantly increased in STNx rats. And Ad‐renalase treatment significantly decreased the phosphorylation of ERK‐1/2 compared with Ad‐β‐gal‐treated rats. However, there is no significant difference in the expression of phosphorylated p38 (p‐p38) among the three groups (Fig. 7B).

## Discussion

In a pathological circumstance such as CRS, bidirectional heart–kidney interactions could lead to a vicious cycle which must be prevented. It has been identified that several hormones or cytokines secreted by kidney or heart have beneficial effect on other organ and may represent as potential therapeutic targets. For instance, EPO prevented cardiac remodelling in nephrectomized rats beyond haematopoiesis and independent of kidney function 37, 38. Conversely, overexpression of hormones such as brain natriuretic peptide 29 and Follistatin‐like 1 (Fstl‐1) secreted by heart, attenuated renal fibrosis and cardiac‐specific Fstl‐1 knockout mice exhibited exacerbation of renal injury after subtotal nephrectomy 39. In the present study, we confirmed the beneficial effects of renalase supplementation on functional and histological alterations of kidney and heart and investigated the underlying mechanism in a rat model with progressive renal injury. We demonstrated that systemic delivery of renalase, a kidney‐derived protein, attenuated both renal and cardiac fibrosis, improved renal function and prevented cardiac remodelling 6 weeks after renal ablation through its anti‐inflammatory and anti‐oxidant effects and inhibition of ERK pathway, indicating that renalase may play a crucial role in the interaction between kidney and heart.

Renalase is a novel protein secreted by kidney and firstly reported to be a catecholamine‐metabolizing enzyme. Our previous reports demonstrated that only kidney tubular epithelial cells could secrete renalase *in vitro*, thus tubular epithelial cells may be the primary source of circulation renalase 40. Reduction of plasma and kidney renalase levels may contribute to elevated circulating catecholamines and consequent SNS activation in CKD rats. In agreement with Baraka's study 41, we found that renalase supplementation indeed decreased the circulation norepinephrine level, SBP and attenuated cardiac hypertrophy. More importantly, rats with renalase administration also showed improvement of albuminuria, glomerulosclerosis, tubular dilation and interstitial fibrosis after renal ablation, which was not observed in previous report 41. This difference may be caused by distinct methods of renalase supplementation. Recombinant protein subcutaneously injection is susceptible to be degraded while adenovirus can broadly infect various cells and leads to effective gene overexpression, is thought to be a more appropriate tool for long‐term experiments. These findings indicated that Ad‐renalase‐administered rats had a lesser degree of CRS; regulation of blood pressure by degrading circulation norepinephrine may be at least in part mechanism for its protective effects.

Inflammation and oxidative stress plays a pivotal role in the progression of CKD. Our recent reports had shown that renalase protected HK2 cells against the cytotoxicity of H_2_O_2_ through suppressing oxidative stress and apoptosis and recombinant renalase protected against contrast‐induced nephropathy through anti‐oxidation and anti‐inflammation mechanism 12, 13. Other reports demonstrated that renalase‐knockout mice led to exacerbation of renal injury caused by acute cisplatin nephrotoxicity and ischemia/reperfusion damage through up‐regulation of pro‐inflammatory cytokines 15, 23. Herein, we found that renalase supplementation also inhibited inflammatory response in remnant kidney *via* down‐regualtion of pro‐inflammatory cytokines and NADPH oxidase components. Besides, we observed that Ad‐renalase treatment could not only reduce macrophage infiltration but also regulate change in macrophage M1/M2 phenotype during CKD progression. Renalase might attenuate renal inflammation *via* inhibiting pro‐inflammatory M1‐like macrophage polarization and promoting anti‐inflammation or antifibrosis M2‐like macrophage activation.

Evidence showed that renalase‐knockout mice exhibit mild LV hypertrophy and renalase polymorphisms are associated with cardiac hypertrophy in patients. Our results proved that renalase supplementation ameliorated cardiac fibrosis and cardiomyocytes hypertrophy, decreased LVPWd and LVEDP and restored cardiac diastolic function in CKD rats. SBP decline after renalase supplementation might be mainly accounted for improvement of cardiac function. Beside, this amelioration was accompanied by remarkably milder cardiomyocytes hypertrophy, reduced expression of pro‐fibrotic markers and inhibition of ERK‐1/2 activation.

It has been established that mitogen‐activated protein kinase (MAPK) activity plays an important role in cardiomyocytes hypertrophy and pressure‐overload induced cardiac remodelling. Takahashi *et al*. 42 reported that the phosphorylation of ERK‐1/2, not p38 MAPK, was involved as an intracellular signalling pathway leading to cardiac hypertrophy in STNx rat model and blockade of ERK‐1/2 phosphorylation could prevent cardiac remodelling, which is consistent with our results. On the contrary, other studies 43, 44 argued that a calcimimetic agent R‐568 or calcitriol increased the activation of ERK‐1/2 and improved cardiac fibrosis 43, 44. The opposite results may be caused by the different duration of experiment design and heterogeneity of animal operation. Interestingly, recent studies showed that phosphorylation of ERK may cause excess oxidative stress which could lead to cardiofibroblasts activation; and apocynin, an inhibitor of NADPH oxidase, attenuated cardiac fibrosis through inhibition ERK1/2 activation in STNx rats 45. We believed that suppressed phosphorylation of ERK‐1/2 after Ad‐renalase treatment was mediated *via* its indirect effects because previous studies revealed that renalase can activate ERK and p38 activation *in vitro*. Renalase treatment had changed the phenotype of STNx animals through decline of blood pressure and sympathetic hyperactivity, which might indirectly inhibit phosphorylation of ERK‐1/2.

Despite of the enzymatic activity of renalase, it has been suggested that renalase is also a cytokine which may mediate its cardio‐renal protective properties through its plasma membrane receptor. PMCA4b, as a recently identified receptor for renalase 22, is a calcium pump that participates in Ca^2+^‐dependent signalling. Previous reports demonstrated that PMCA4b transgenic mice exhibited improvement of LV hypertrophy following transverse aortic constriction or phenylephrine/Ang II infusion and PMCA4b overexpression inhibited cardiomyocyte hypertrophy *in vitro* 46. Supplementation of renalase may directly antagonize cardiac hypertrophy through activating extracellular PMCA4b on cardiomyocytes and related downstream signalling pathway in an endocrine manner.

There are some limitations of this study. First, the study duration is only 6 weeks which may be too short for CKD animals. Second, we could not conclude that renalase exerts its protective effects by lowering blood pressure or directly inhibiting fibrosis and hypertrophy of hearts because of lack of *in vitro* studies. Thus, kidney‐specific renalase‐knockout animal should be used in future study to determine the mechanism of renalase's protective effects in type 4 CRS.

In summary, renalase supplementation by systemic delivery of adenovirus‐renalase ameliorated both renal dysfunction and cardiac remodelling after subtotal nephrectomy in catecholamine‐lowering‐dependent/independent manners. The potential mechanism of its protective effects may be mediated by its anti‐inflammatory, anti‐oxidant function and inhibition of ERK1/2 pathway. Therefore, renalase is a crucial modulator of CRS progression and renalase supplementation may be a promising therapy for prevention and deterioration of CRS in CKD patients.

## Conflicts of interest

None.

## Supporting information


**Figure S1** Adenovirus‐mediated renalase expression efficacy *in vivo*.

**Figure S2** Evaluation of renalase expression efficacy by immunostaining.

**Figure S3** Reprehensive echocardiography at week 6.

**Table S1** Primer sequences used in real‐time PCR.
