# Supplementary figures and images for: Renalase attenuates hypertension, renal injury and cardiac remodelling in rats with subtotal nephrectomy
Source: J Cell Mol Med. 2016 Feb 29;20(6):1106–17. doi: 10.1111/jcmm.12813 (PMC4882988; doi:10.1111/jcmm.12813)

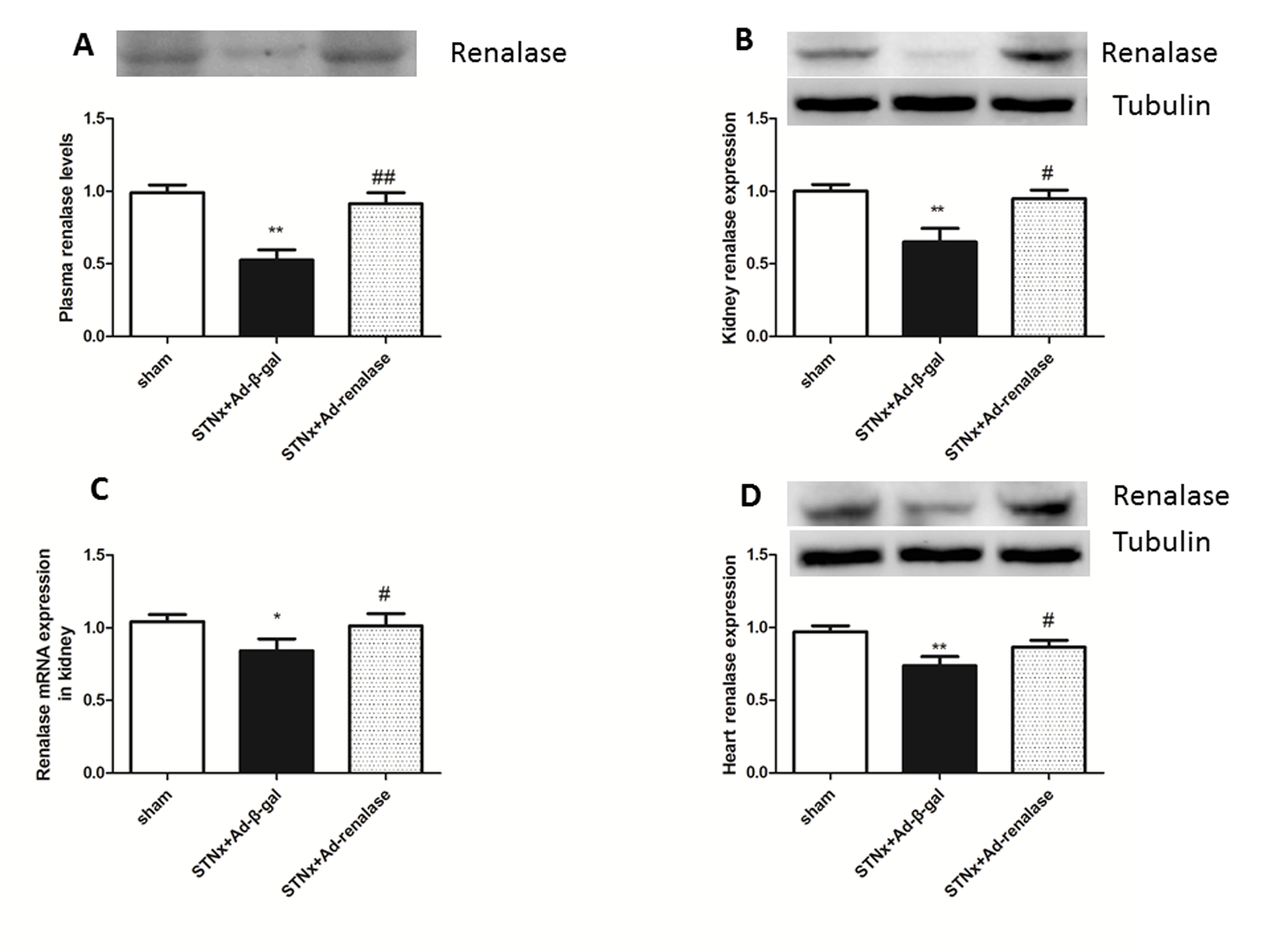

Supplement: Supplementary file 1 — Figure S1 Adenovirus‐mediated renalase expression efficacy in vivo. [file JCMM-20-1106-s001.tif]

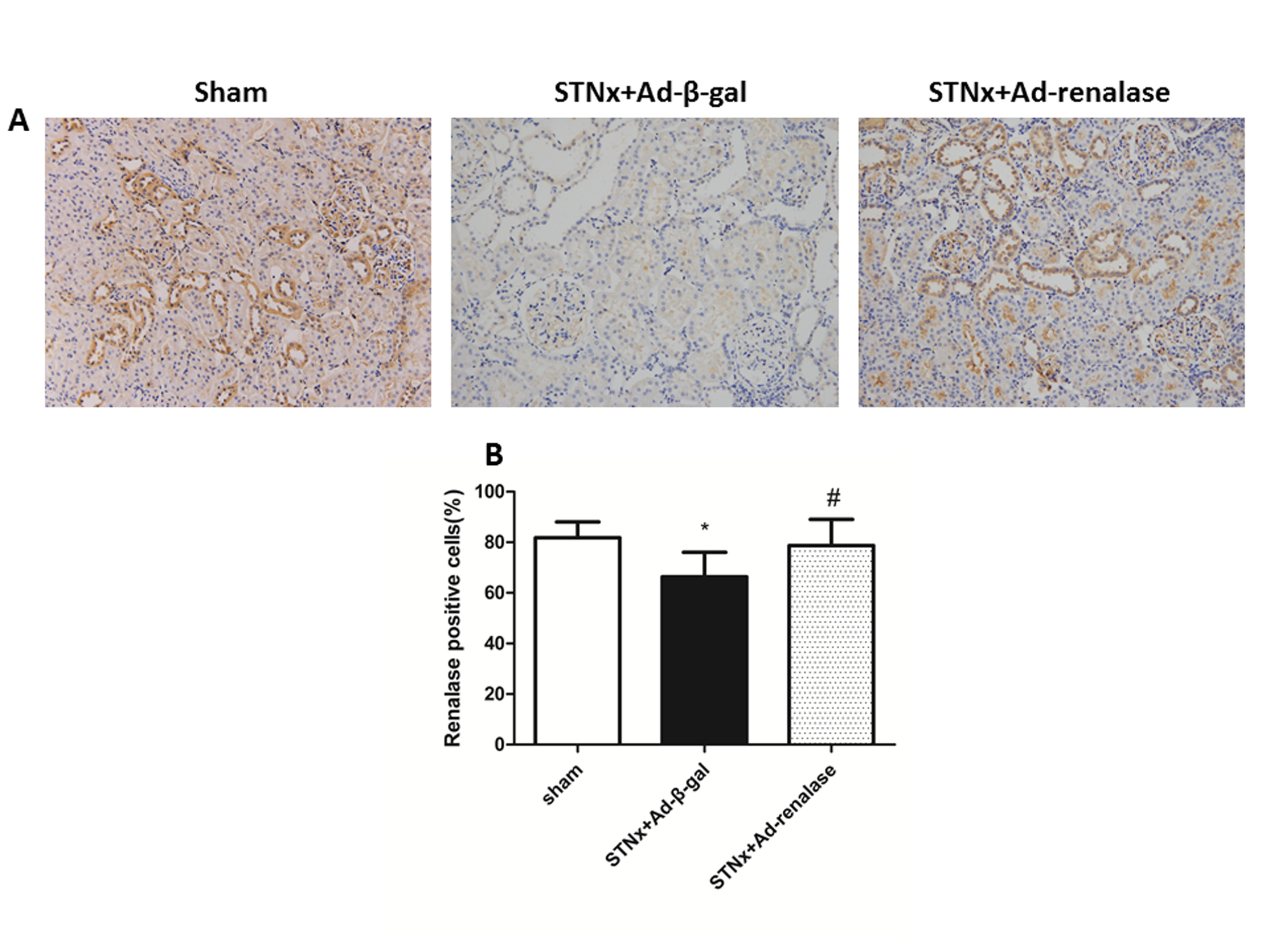

Supplement: Supplementary file 2 — Figure S2 Evaluation of renalase expression efficacy by immunostaining. [file JCMM-20-1106-s002.tif]

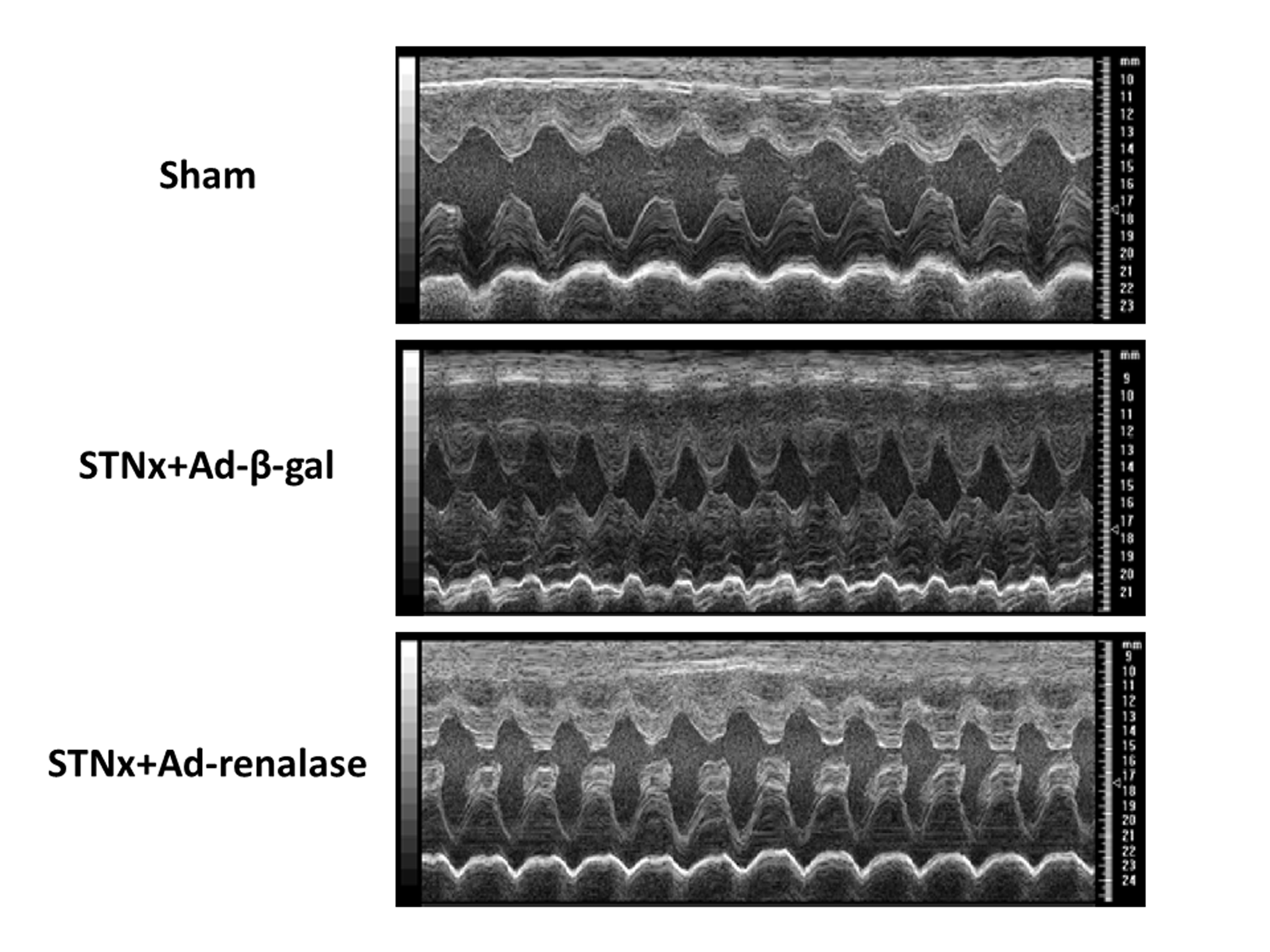

Supplement: Supplementary file 3 — Figure S3 Reprehensive echocardiography at week 6. [file JCMM-20-1106-s003.TIF]
