# Supplementary material for: Renalase attenuates hypertension, renal injury and cardiac remodelling in rats with subtotal nephrectomy
Source: J Cell Mol Med. 2016 Feb 29;20(6):1106–17. doi: 10.1111/jcmm.12813 (PMC4882988; doi:10.1111/jcmm.12813)
Supplement: Supplementary file 4 — Table S1 Primer sequences used in real‐time PCR. [file JCMM-20-1106-s004.docx]

**Supporting information**

**Table 1**. Primer sequences used in real-time PCR

| Gene | Primer sequence |
| --- | --- |
| renalase | Forward: 5’ GAAAAATCATTGCAGCCTCTCA 3’ |
|  | Reverse: 5’ AAGTTCTGCCTGTGCCTGTGTA 3’ |
| TGF-β1 | Forward: 5’ CCAGCCGCGGGACTCT 3’ |
|  | Reverse: 5’ TTCCGTTTCACCAGCTCCAT 3’ |
| Collagen I | Forward: 5‘ ATCCTGCCGATGTCGCTAT’ 3’ |
|  | Reverse: 5’ CCACAAGCGTGCTGTAGGT 3’ |
| Collagen III | Forward: 5’ CTGGTCCTGTTGGTCCATCT 3’ |
|  | Reverse: 5’ ACCTTTGTCACCTCGTGGAC 3’ |
| TNF-α | Forward: 5’ GTCTGTGCCTCAGCCTCTTC 3’ |
|  | Reverse: 5’ TGGAACTGATGAGAGGGAGC 3’ |
| IL-6 | Forward: 5’ TCTGGAGTTCCGTTTCTACCTGG 3’ |
|  | Reverse: 5’ CATAGCACACTAGGTTTGCCGAG 3’ |
| MCP-1 | Forward: 5’ TGAACTTGACCCATAAATC 3’ |
|  | Reverse: 5’ TAGGGAGGAATAGTGTAAT 3’ |
| gp91^phox^ | Forward: 5’ TGCTGCTCGA ATATGAATGG 3’ |
|  | Reverse: 5’ TGATGGC TTCCAGCAACTC 3’ |
| p47^phox^ | Forward: 5’ CAG AAT GTT GCC TGG TTG 3’ |
|  | Reverse: 5’ GTG CCC TCC CTT AGA TGA 3’ |
| p67^phox^ | Forward: 5’ TACTTCCAACGAGGGATGCTC 3’ |
|  | Reverse: 5’ AGCTTTCCTCCTGGGGCT 3’ |
| GAPDH | Forward: 5’ ACTCCACGACATACTCAGCA 3’ |
|  | Reverse: 5’ CATCAACGACCCCTCATT 3’ |


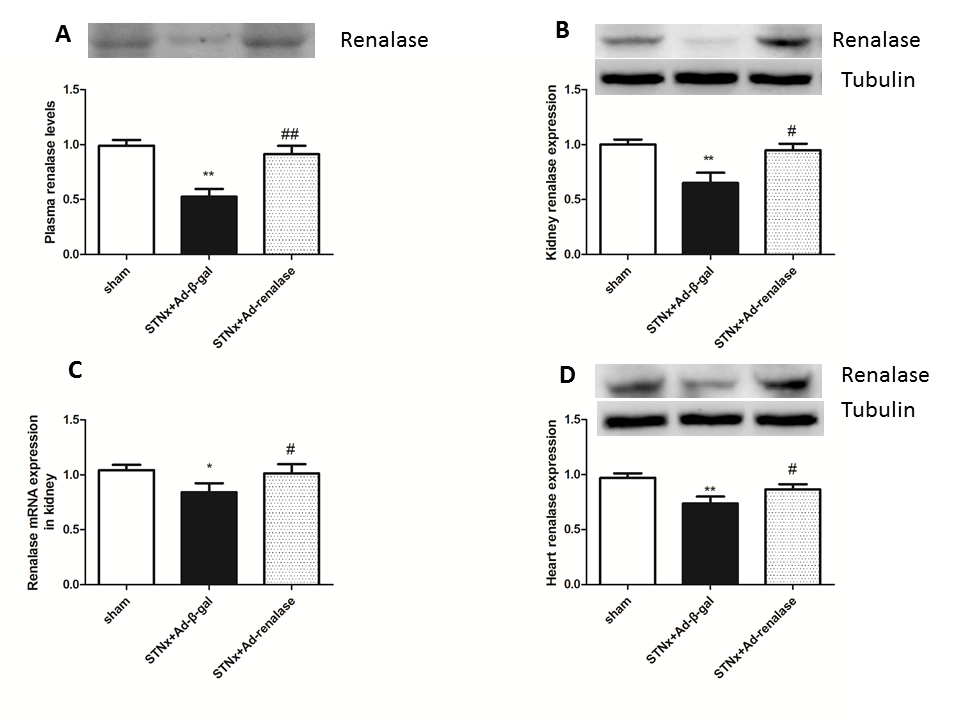


**Fig. 1** Adenovirus-mediated renalase expression efficacy *in vivo.* Representative western blots for renalase expression and relative quantification in plasma (A) , kidney (B) and heart tissue (B) in sham-operated, Ad-β-gal-treated rats and Ad-renalase-treated rats. Relative kidneymRNA expression of renalase (D) was calculated. All values are presented as means$\pm$SEM (n=10). *^*^P*﹤0.05 *versus* sham; ^#^*P*﹤0.05 *versus* STNx+Ad-β-gal;^**^*P*<0.01 versus sham; ^##^*P*<0.01 versus STNx+Ad-β-gal.

**
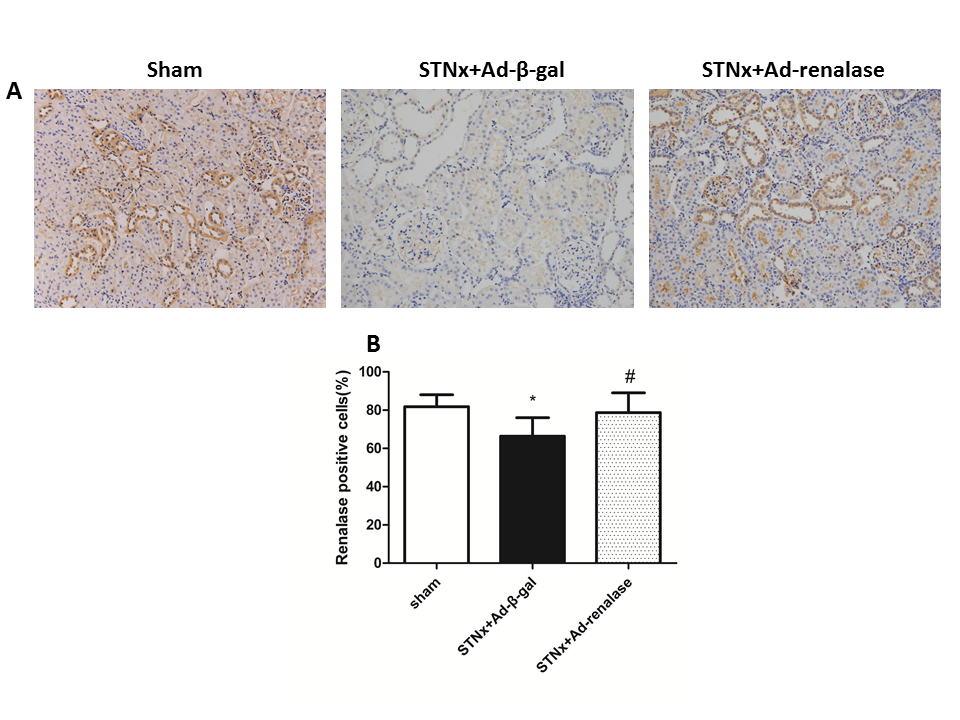
**

**Fig. 2** Evaluation of renalase expression efficacy by immunostaining. (A)Representative renalase staining images from left kidney in three groups, Magnification, ×200. (B) Quantitative analysis of percentage of renalase positive cells. All values are presented as means$\pm$SEM (n=10).*^*^P*﹤0.05 versus sham; ^#^*P*﹤0.05 versus STNx+Ad-β-gal.


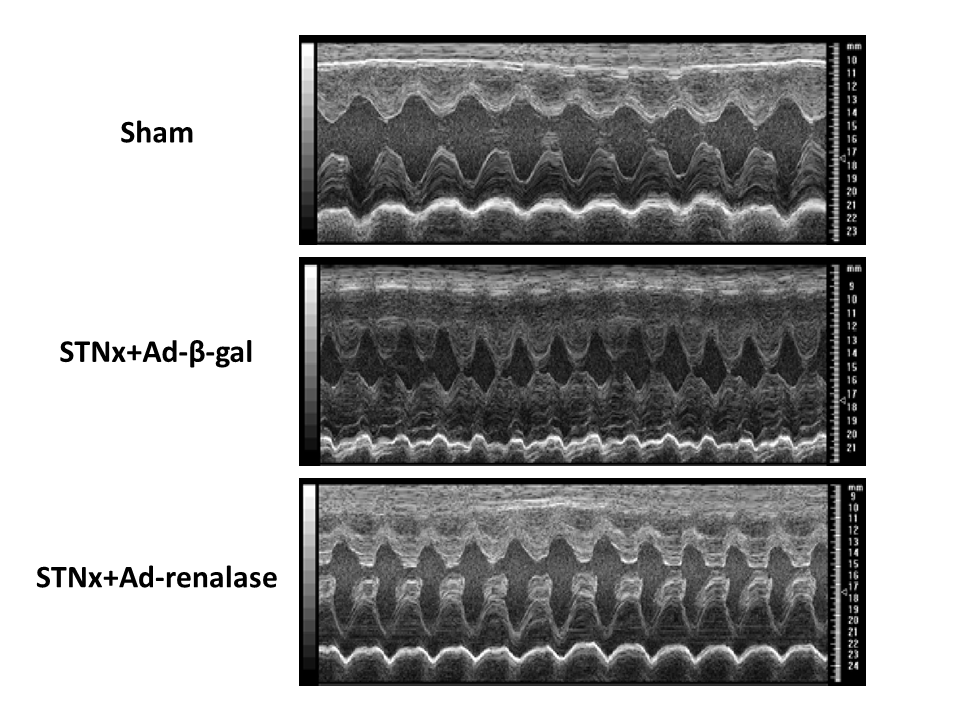


**Fig. 3** Echocardiography at week 6. Reprehensive photographs of parasternal short axis view in M mode of sham, Ad-β-gal -treated STNx and Ad-renalase-treated STNx rats.
